# Whole Exome Sequencing Identifies Novel Genes for Fetal Hemoglobin Response to Hydroxyurea in Children with Sickle Cell Anemia

**DOI:** 10.1371/journal.pone.0110740

**Published:** 2014-10-31

**Authors:** Vivien A. Sheehan, Jacy R. Crosby, Aniko Sabo, Nicole A. Mortier, Thad A. Howard, Donna M. Muzny, Shannon Dugan-Perez, Banu Aygun, Kerri A. Nottage, Eric Boerwinkle, Richard A. Gibbs, Russell E. Ware, Jonathan M. Flanagan

**Affiliations:** 1 Hematology Center, Department of Pediatrics, Baylor College of Medicine, Houston, Texas, United States of America; 2 The University of Texas Graduate School of Biomedical Sciences at Houston, Department of Biostatistics, Bioinformatics, and Systems Biology, University of Texas, Houston, Texas, United States of America; 3 Human Genetics Center, University of Texas, Houston, Texas, United States of America; 4 Human Genome Sequencing Center, Baylor College of Medicine, Houston, Texas, United States of America; 5 Division of Hematology, Cincinnati Children's Hospital Medical Center, Cincinnati, Ohio, United States of America; 6 Steven and Alexandra Cohen Children's Medical Center of New York, New Hyde Park, New York, United States of America; 7 Department of Hematology, St. Jude Children's Research Hospital, Memphis, Tennessee, United States of America; Emory University/Georgia Insititute of Technology, United States of America

## Abstract

Hydroxyurea has proven efficacy in children and adults with sickle cell anemia (SCA), but with considerable inter-individual variability in the amount of fetal hemoglobin (HbF) produced. Sibling and twin studies indicate that some of that drug response variation is heritable. To test the hypothesis that genetic modifiers influence pharmacological induction of HbF, we investigated phenotype-genotype associations using whole exome sequencing of children with SCA treated prospectively with hydroxyurea to maximum tolerated dose (MTD). We analyzed 171 unrelated patients enrolled in two prospective clinical trials, all treated with dose escalation to MTD. We examined two MTD drug response phenotypes: HbF (final %HbF minus baseline %HbF), and final %HbF. Analyzing individual genetic variants, we identified multiple low frequency and common variants associated with HbF induction by hydroxyurea. A validation cohort of 130 pediatric sickle cell patients treated to MTD with hydroxyurea was genotyped for 13 non-synonymous variants with the strongest association with HbF response to hydroxyurea in the discovery cohort. A coding variant in *Spalt-like transcription factor*, or *SALL2*, was associated with higher final HbF in this second independent replication sample and *SALL2* represents an outstanding novel candidate gene for further investigation. These findings may help focus future functional studies and provide new insights into the pharmacological HbF upregulation by hydroxyurea in patients with SCA.

## Introduction

Sickle cell anemia (SCA) is an inherited blood disorder, affecting 1 in 400 African Americans, causing significant morbidity and mortality. Although a monogenic disease, individuals with SCA (usually homozygous HbSS) exhibit wide variability in their laboratory and clinical phenotypes. One of the most powerful and reproducible modifiers of disease severity is an individual's endogenous level of fetal hemoglobin (HbF) [1]. If produced in sufficient amounts, HbF is able to prevent the intracellular polymerization of deoxygenated sickle hemoglobin (HbS), which is the nidus of the clinical disease process [2], [3]. Pharmacologic induction of HbF is clinically beneficial, and the most widely used and safest method for increasing HbF levels in patients with SCA is treatment with hydroxyurea. Currently, it is the only FDA-approved pharmacologic treatment for induction of HbF in adult patients with SCA, and is approved by the European Medicines Agency for both children and adults with SCA. Hydroxyurea significantly reduces pain and acute chest episodes, the need for blood transfusions and hospitalizations, and most importantly, reduces mortality [4]–[6]. While hydroxyurea has suspected disease modulating effects outside of HbF induction, the majority of its benefit is directly related to the amount of HbF produced in response to the drug [7], [8]. There is an inverse relationship between levels of drug-induced HbF and number of pain episodes, hospitalizations, and overall mortality [9], [10].

Several clinical studies have shown that individual hematological responses to hydroxyurea treatment are highly variable, with induced HbF levels ranging from 10% to greater than 30% HbF even for compliant patients on similar dosing regimens [11]–[14]. Previous efforts to identify predictors associated with final HbF produced in response to hydroxyurea have identified higher baseline HbF values, higher white blood cell count (WBC), and absolute reticulocyte count (ARC) as important factors [13], [15], [16]. However, none of these parameters can accurately predict the degree of HbF induction by hydroxyurea in an individual patient. From analysis of sibling pairs, it is known that the degree of HbF induction by hydroxyurea has a strong heritable component [17], indicating that genetic modifiers may have a large effect on drug response. Identification of specific genetic variants associated with HbF induction may elucidate reasons for this phenotypic variability and provide new insights into the drug's mechanisms of action related to HbF induction.

The aim of this study was to use a whole exome sequencing (WES) pharmacogenomics approach to identify genetic predictors of HbF response to hydroxyurea. Using two prospective pediatric cohorts with robust HbF phenotype data and standardized dose escalation regimen to MTD as a discovery cohort, we undertook a novel unbiased screen to test the entire exome for variants that are associated with hydroxyurea-induced HbF response levels (as measured by maximum %HbF at MTD [final HbF] or the change in %HbF from baseline to final [ΔHbF]). We focused on genetic variants with predicted functional effects on protein coding regions and identified several non-synonymous mutations that may influence the HbF response to hydroxyurea in children with SCA. We then validated a coding variant in *SALL2* in an unrelated, “real-world” cohort of children treated with hydroxyurea.

## Results

### Patient characteristics

Overall, both cohorts showed robust response to hydroxyurea with evidence of substantial individual variability in drug response (Table 1). All discovery cohort samples were genotyped for variants in *BCL11A* (rs1427407, rs4671393, rs11886868, rs7599488) and *HBSIL-MYB* (rs9399137, rs9402686); we found an association with baseline HbF for all *BCL11A* variants tested other than rs7599488. There was no significant association between the *BCL11A* variants tested and final HbF. No association with baseline, final or HbF was seen for either *HBSIL-MYB* variants tested. Linear association was performed with *BCL11A* variants as a covariate, without a significant change in associations.

**Table 1:** Comparison of discovery and validation cohorts.

	Discovery Cohort		Validation Cohort		Discovery Cohort		Validation Cohort	
**Age (years)**	10.4	±4.5	8.1	±4.0		-		-
**WBC (x10^9^/L)**	13.5	±4.2	13.1	±5.7	6.5	±2.0	6.9	±2.0
**ANC (x10^9^/L)**	7.2	±3.2	6.2	±4.3	3.0	±1.2	2.7	±0.9
**Hb (g/dL)**	9.1	±0.9	8.0	±1.7	9.4	±1.0	9.5	±1.3
**ARC (x10^9^/L)**	0.28	±0.13	0.29	±0.12	0.17	±0.09	0.12	±0.05
**MCV (fL)**	86.1	±4.8	82.1	±8.3	116.4	±13.0	97.7	±11.1
**HbF (%)**	8.0	±4.9	11.9	±5.9	27.6	±7.3	25.8	±8.2
**ΔHbF (%)**		-		-	19.5	±6.6	13.9	+7.0
**HU dose (mg/kg/day)**		-		-	25.1	±4.5	27.1	±4.3

WBC: white blood cell count; ANC: absolute neutrophil count; ARC: absolute reticulocyte count; MCV: mean corpuscular volume; HU: hydroxyurea.

The discovery cohort was composed of 120 patients from HUSTLE and 51 from SWiTCH. The validation cohort was collected from patients treated at TCCH.

**Figure 1:**
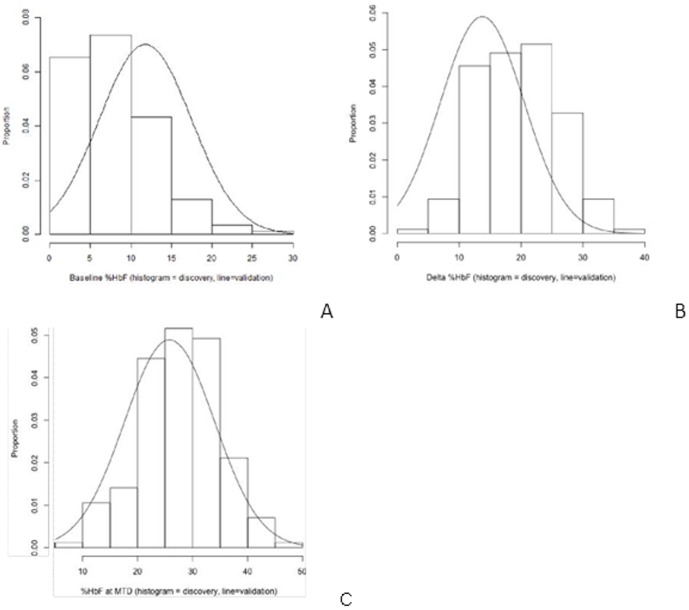
Comparison of discovery and validation cohorts. A, Baseline, or endogenous HbF for the discovery cohort is shown in binned histogram, and distribution of baseline HbF in validation cohort by a line plot. B, Delta HbF for the discovery cohort is shown in binned histogram, and distribution of delta HbF in validation cohort by a line plot. C, Final, or MID HbF for the discovery cohort is shown in binned histogram, and distribution of final, or MID in validation cohort by a line plot.

At the time of hydroxyurea initiation, the average age of the 171 patients in the discovery cohort was 10.4±4.5 years of age. The average age of the 130 patients in the validation cohort was 8.1±4.0 years. All patients were treated under a similar dose escalation to MTD regimen according to protocol, or similar institutional guidelines 18, 19. After a minimum of 6 months on hydroxyurea therapy, all patients reached a stable MTD (average 25.1±4.5 mg/kg/day in the discovery cohort, 27.1±4.3 mg/kg/day in the validation cohort) with predictable laboratory benefits (Table 1). The mean increase in HbF was 19.5±6.6% in the discovery cohort and 13.9±7.0% in the validation cohort, reflecting slight differences between the two groups of patients. There was evidence of consistent myelosuppression across both cohorts, however. The baseline HbF, distribution of ΔHbF at MTD and final HbF at MTD were all similar to prior reports (Figure 1, A–C). [15], [20]

### Whole exome sequencing

All 171 samples in the discovery cohort passed stringent WES quality control parameters with an average of 92% of the targeted exonic regions sequenced at greater than 20× coverage per individual. We identified a total of 278,639 autosomal variants, and 127,238 of these variants were non-synonymous or splice site variants expected to introduce an amino acid change in their encoded proteins (Table S1). For single variant association testing, we further filtered the non-synonymous variants (n = 127,238) for those with a minor allele frequency (MAF) greater than or equal to 2% (n = 38,012). We corrected for any population stratification using principal component analysis (PCA) performed by the EIGENSTRAT method.

As our phenotypes of interest are continuous variables, we used linear regression analysis to test the association of the 38,012 common (MAF≥2%) non-synonymous variants using final HbF, and ΔHbF as independent, continuous variables. In addition, we attempted to find rare variants with MAF<2% associated with drug response by performing burden analysis with SKAT and T2 tests using the ΔHbF and final HbF phenotypes, but none of the gene level p-values were significant.

We identified 12 variants associated with ΔHbF with a p-value less than 5×10^−4^ (Table 2). In addition, we identified 13 variants associated with final HbF, also with a p-value less than 5×10^−4^ (Table 3). Although none of the p-values achieved genome wide significance level (p<1.3X10^−6^), these results offered suggestive signals of potential associations. We used the existing methods of SIFT and PolyPhen2 [21] for predicting the functional impact of each non-synonymous variant to estimate which of the 25 variants had a predicted damaging or benign effect on encoded protein function (Tables 2 and 3).

**Table 2:** Variants associated with ΔHbF on hydroxyurea.

Gene	SNP ID	Amino Acid Change	Protein Function Prediction	MAF (%)	Function	Beta Value	SE	P-value
***EML1***	rs141631682	Gly109Asp	Damaging	2	Microtubule assembly	10.9	2.7	7.41×10^−5^
***SESN1***	rs2273668	Leu103Ile	Damaging	2	Peroxiredoxin reduction	9.3	2.3	8.88×10^−5^
***PAPLN***	rs17126352	Val416Ile	Benign	3	Metalloprotease	7.1	1.8	1.35×10^−4^
***DCHS2***	rs61746132	Pro1676Lys	Damaging	12	Calcium dependent cell adhesion	3.9	1.0	1.73×10^−4^
***KRT80***	rs61749462	Arg364Ser	Damaging	3	Keratin 80	8.0	2.1	1.80×10^−4^
***SALL2***	rs61743453	Pro840Arg	Damaging	4	Transcription factor	6.7	1.8	2.37×10^−4^
***NOM1***	rs61742645	Arg779Cys	Benign	2	PP1 interacting protein	8.7	2.3	2.43×10^−4^
***N4BP2L2***	rs35108810	Asp246Val	Damaging	15	*ELA2* transcription inhibitor	−3.3	0.9	3.47×10^−4^
***RNF113B***	rs16955011	Val92Met	Damaging	18	Ring finger protein	3.1	0.8	3.76×10^−4^
***FTSJ2***	rs55904231	Ser11Phe	Benign	7	RNA methyltransferase	5.2	1.4	3.76×10^−4^
***RHPN2***	rs28626308	Arg70Gln	Damaging	7	Rho GTPase binding protein	5.2	1.4	3.91×10^−4^
***ADAR***	rs17843865	Tyr587Cys	Benign	4	Adenosine deaminase	−6.4	1.8	4.65×10^−4^

Variants selected were predicted damaging, with a p-value <0.001 in the discovery cohort, n = 171, composed of patients from HUSTLE and SWiTCH trials.

**Table 3:** Variants associated with final HbF on hydroxyurea.

Gene	SNP ID	Amino Acid Change	Protein Function Prediction	MAF (%)	Function	Beta Value	SE	P-value
***RSPH3***	rs61750777	Ala154Val	Damaging	6	Radial spoke protein	−6.5	1.6	6.55×10^−5^
***OLR1***	rs11053646	Lys167Asn	Benign	2	Opioid receptor	3.4	0.9	1.22×10^−4^
***SEC31B***	rs11819496	Arg478Thr	Damaging	2	ER transport	−12.0	3.1	1.55×10^−4^
***COPE***	rs34510432	Arg85 His	Damaging	2	ER transport	9.9	2.6	1.88×10^−4^
***RNF113B***	rs16955011	Val92Met	Damaging	18	Ring finger protein	3.4	0.9	1.93×10^−4^
***CDHR3***	rs6967330	Cys529Tyr	Benign	29	Calcium dependent cell adhesion	3.1	0.8	2.58×10^−4^
***ETAA1***	rs3770655	Pro771Ser	Benign	89	Ewing's tumor associated antigen	4.3	1.2	2.84×10^−4^
***TTLL10***	rs113596156	Arg35Gln	Benign	2	Polyglycylase	9.8	2.7	2.91×10^−4^
***DOCK1***	rs869801	Ala1793Thr	Benign	21	Cytokinesis	−3.5	1.0	3.60×10^−4^
***MYBBP1A***	rs899441	Lys637Glu	Benign	6	MYB associated	−5.4	1.5	4.26×10^−4^
***MARCH10***	rs116835087	Gly587Ser	Damaging	6	Membrane-associated ring finger	−6.0	1.7	4.33×10^−4^
***PKD1L1***	rs11972142	Thr879Ala	Benign	15	Polycystic kidney disease like	3.6	1.0	4.78×10^−4^
***ADCY10***	rs16859886	Thr234Met	Benign	12	Adenyl cyclase	3.9	1.1	4.96×10^−4^

Variants selected were predicted damaging, with a p-value <0.001 in the discovery cohort, n = 171, composed of patients from HUSTLE and SWiTCH trials.

From these 25 variants, we identified 13 variants with strongest association with response to hydroxyurea and predicted to introduce an amino acid change that has a damaging effect on protein structure or function. We genotyped these 13 variants by TaqMan PCR in our independent validation cohort of 130 patients with SCA. We found that one of the 13 variants, located in the *SALL2* gene, maintained association with hydroxyurea response. In the discovery cohort, the P840R variant in the *SALL2* gene (rs61743453) was associated with a higher change in HbF in response to hydroxyurea (p = 2.37×10^−4^, beta value 6.7). In the validation cohort, this same P840R variant was associated with a higher final HbF, with a p-value of 0.05, and a beta value of 4.2. Using Fisher's combined probability test method, a meta analysis of the association of the *SALL2* variant with ΔHbF in the discovery and validation sample groups (n = 301) leads to a combined p-value of 8.30×10^−4^. The meta analysis of the association of *SALL2* with final HbF in the discovery and validation sample groups resulted in a combined p-value of 1.48×10^−4^.

## Discussion

Many individuals with SCA are prescribed hydroxyurea, and there is evidence that genetic modifiers affect individual response [17], [22]. In order to identify novel candidate genes and variants associated with hydroxyurea response, we sequenced the exomes of 171 individuals enrolled in two prospective clinical trials and related their sequence variant data to HbF response. This discovery cohort was obtained from patients treated on protocol, with the highest level of drug compliance supervision, including monthly pill counts. Our validation cohort (n = 130) was treated under guidelines similar to that of the discovery cohort. As expected, individual MTD was achieved at different hydroxyurea doses, within a range of 10–35 mg/kg/d, reflecting the typical range in bioavailability among patients. There was no correlation between hydroxyurea dose and HbF response (p = 0.56), supporting the conclusion that differences in pharmacokinetics and pharmacodynamics affect HbF levels achieved on hydroxyurea [22].

Whole exome sequencing permitted analysis of genes beyond a usual set of *a priori* biologic candidate genes for this phenotype and variants across a broad allele frequency spectrum. We identified multiple non-synonymous variants associated with ΔHbF or final HbF (Tables 2 and 3) in the discovery cohort. We then performed genotyping on a validation cohort for 13 candidate variants with the lowest p-values and were also predicted to be damaging. Of the 13 variants genotyped, the variant in SALL2 was associated with a higher HbF in the discovery cohort, and a higher final HbF at MTD in the discovery cohort. The validated variant in *SALL2* represents a novel variant not previously implicated in -globin expression or HbF response to hydroxyurea. Further studies of other sickle cell cohorts treated with hydroxyurea are needed to confirm this promising association.

**Figure 2:**
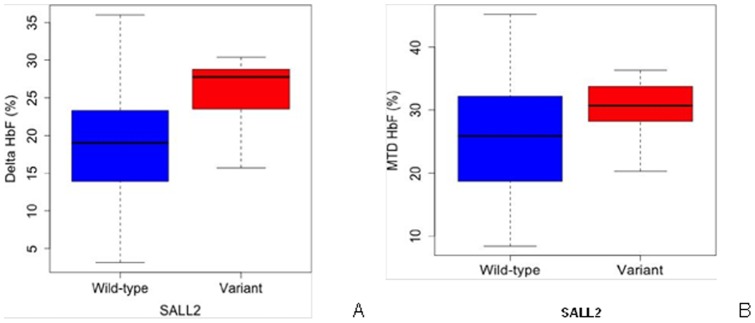
Effect of *SALL2* variant rs61743453 on HbF response to hydroxyurea. A, Effect of rs61743453 on delta HbF in discovery cohort. B, Effect of rs61743453 on MTD HbF in validation cohort. Variant refers to the Pro840Arg variant; no individuals were homozygous for this change.

SALL2 is a multi-zinc finger transcription factor implicated in hematopoietic cell maturation and cell cycle arrest [23]. It contains the same conserved 12 amino acid N-terminal motif as BCL11A [24], [25]. This motif has been shown to be essential for binding of the nucleosome remodeling and deacetylase co-repressor complex, or NuRD, which includes the histone deacelylases HDAC1 and HDAC2. Both HDAC1 and HDAC2 have been shown to act as co-repressors of gamma globin [26]. The variant identified here (rs61743453), causes a proline to arginine change at residue 840 and is predicted to bedamaging to protein function. This P840R SALL2 variant was associated with a higher HbF response to hydroxyurea (Figure 2). Further functional studies will help establish the role of SALL2 in HbF induction in the context of hydroxyurea.

Despite the relatively small sample size, we used the best genotype-phenotype pairs available from prospectively treated pediatric patients from two clinical trials for the discovery cohort. We assembled a validation cohort from patients treated with hydroxyurea according to standard of care and expert guidelines in a pediatric hematology center with an established sickle cell program.

This study may have failed to detect loci with modest effects because of low statistical power and some associations identified in the discovery cohort may have failed validation given the small size of the validation cohort. Accordingly, all of the mutations identified in the discovery cohort may represent coding variants worth pursuing in future studies. The data presented here bodes well for the success of larger collaborative efforts aimed at identifying genetic modifiers of hydroxyurea response, and serves as a call for coordinated collaboration among pediatric sickle cell centers to increase sample size and increase the odds for novel discovery and translational potential.

## Methods

### Subjects

The discovery cohort was composed of 171 unrelated children with SCA; 120 were enrolled in the Hydroxyurea Study of Long-Term Effects (HUSTLE, NCT00305175); and 51 were enrolled in the NHLBI-sponsored Stroke with Transfusions Changing to Hydroxyurea (SWiTCH, NCT00122980). HUSTLE was a single center trial investigating long term effects of hydroxyurea in SCA, while SWiTCH was a multi-center trial investigating the use of hydroxyurea on stroke prevention. HUSTLE and SWiTCH study patient samples were used with approval from the Baylor College of Medicine Internal Review Board, protocol H-29047. Patients and their families in both clinical trials provided written consent for DNA sample collection, storage and sequencing. Texas Children's Hospital Hematology Center patients and their families provided written consent to whole exome sequencing, posting of sequences to dbGAP, and data collection under BCM Internal Review Board protocol H31356. All DNA samples and data in this study were denominalized for analysis. All 171 individuals had a known baseline HbF level measured at greater than 3 years of age, were initially treated with hydroxyurea at 20 mg/kg, and then dose-escalated to mild myelosuppression using a standardized regimen [18], [19].

The validation cohort contained 130 unrelated children with SCA followed at the Texas Children's Hospital Hematology Center (TCHHC). All patients receiving hydroxyurea at TCHHC were approached for enrollment in an Internal Review Board-approved protocol for genetic analysis. They were treated with hydroxyurea using institutional guidelines rather than a specific protocol, but all were escalated to MTD following a standardized regimen as previously described [19]. All patients were treated with hydroxyurea for at least 6 months prior to the designated MTD timepoint. Total HbF levels for both discovery and validation cohorts were measured by HPLC.

### Ethics Statement

All patients and their families gave informed consent for genomic DNA sample collection, storage, and sequencing. The WES genomic analyses were approved by the Baylor College of Medicine Institutional Review Board. All DNA samples and data in this study were denominalized for analysis.

### Whole exome sequencing

DNA concentrations were quantified using picogreen fluorescent detection method (Quant-iT, Invitrogen). For each DNA sample, the entire exome was captured using the NimbleGen VCRome 2.1 capture reagent followed by sequencing on an Illumina platform using standard chemistries. The sequencing reads were mapped to Hg19 reference genome using the BWA [27]. Sample level genome variants were identified and annotated using the Human Genome Sequencing Center's integrated Mercury pipeline which includes quality score recalibration and insertion/deletion (Indel) realignment, genome variant identification by AtlasSNP [28], and annotation using Cassandra software. A project level variant call format (VCF) was generated for all the samples, and included variants that were present in at least one sample. Variants with more than 5% missed genotyping calls were excluded from analysis. Error threshold for alignment was two base errors per read with penalties for indels (maximum of 1) are much more costly than the penalties for SNVs (maximum of 2). We allowed for read-trimming to 35 bp.

WES genotyping of variants of interest with heterozygosity scores less than 0.45 were verified by TaqMan genotyping. TaqMan genotyping assays were performed on an Applied Biosystem's StepOne instrument (AB, Foster City, CA). After 40 amplification cycles, threshold cycle values were automatically calculated, and the individual SNP genotypes were called by the StepOne v2.0 software (AB, Foster City, CA).

### Statistical Analysis

Linear regression analysis was used to test the association of the filtered variants using final HbF and ΔHbF as independent, continuous variables. The ΔHbF and final HbF phenotypes both had normal distributions, indicating they were suitable for linear regression analysis (Figure 1); values were adjusted for age and gender. Principal component analysis (PCA) was performed using the EIGENSTRAT method, and applied to all models. Quality control filtering steps, including SNP missingness check, removal of sex chromosomes, monomorphic site, synonymous and intronic variant removal, excess heterozygosity filter and minor allele frequency (MAF) cut-off of 2% and the impact of these filtering steps on the total number of SNPs, are described in Table S1.

To analyze the effect of rare variants (MAF<2%) on the phenotypes ΔHbF and final HbF, we used two gene based tests, a simple burden test (T2) and SKAT [29], [30]. In SKAT and T2 testing, a collection of rare variants within a single gene are tested for association with the phenotype. The T2 considers those rare variants with MAF less than 2% and assumes that the effects of all variants are in the same direction. SKAT is a kernel-based test that considers rare variants having effects in either direction [29], [31]. Both tests considered only non-synonymous variants.

### Validation

Genomic DNA from 130 patients from TCHHC collected as a validation cohort were genotyped by TaqMan or Sanger sequencing for 13 SNPs with the lowest p-values that were identified as associated with HbF response to hydroxyurea, non-synonymous, and damaging. The relationship between genotype and HbF response to hydroxyurea was analyzed with a one directional t-test.

## Supporting Information

Table S1 **Quality control filters used in WES analysis.** Sex chromosomes were removed, as gender did not impact HbF response to hydroxyurea. Sites with heterzygousto homozygous ration>0.4 were removed.Variants with MAF<2% were analyzed by burden testing.
(PDF)
