# Supplementary material for: Whole Exome Sequencing Identifies Novel Genes for Fetal Hemoglobin Response to Hydroxyurea in Children with Sickle Cell Anemia
Source: PLoS One. 2014 Oct 31;9(10):e110740. doi: 10.1371/journal.pone.0110740 (PMC4215999; doi:10.1371/journal.pone.0110740)
Supplement: Table S1 — Quality control filters used in WES analysis. Sex chromosomes were removed, as gender did not impact HbF response to hydroxyurea. Sites with heterzygousto homozygous ration>0.4 were removed.Variants with MAF<2% were analyzed by burden testing. (PDF) [file pone.0110740.s001.pdf]

Quality Control Filtering Step	Number of SNPs	Number of SNPs removed	Number of SNPs remaining
**Total number of identified variants**	278,639	-	-
**Removed sites with > 5% missing genotyping**	278,639	14,242	264,397
**Removal of X and Y chromosome variants**	264,397	6,218	258,179
**Removed synonymous and intronic variants**	258,179	131,744	126,435
**Removed sites without variation**	126,435	2,841	123,594
**Excess heterozygosity filter**	123,594	211	123,383
**Minor Allele Frequency≥0.2**	123,383	83,848	39,535
**Removed variants not predicted to be damaging by PloyPhen2/SIFT**	39,535	26,784	12,751

Table S1: Quality control filters used in WES Analysis. Sex chromosomes were removed, as gender did not impact HbF response to hydroxyurea. Sites with heterozygous to homozygous ratio >0.4 were removed. Variants with MAF<2% were analyzed by burden testing.

Supplemental Figure 1: Distribution of patient HbF Values. The normal distribution of the change in HbF at MTD (A) and final HbF at MTD (b) in our study population is demonstrated.

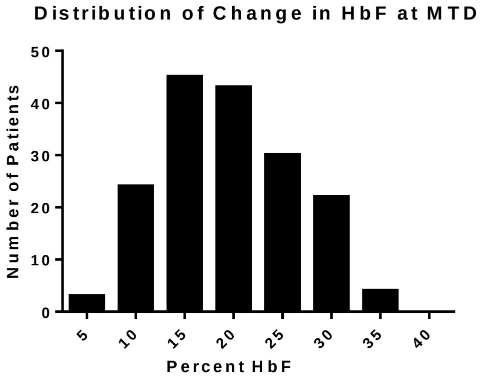

A.

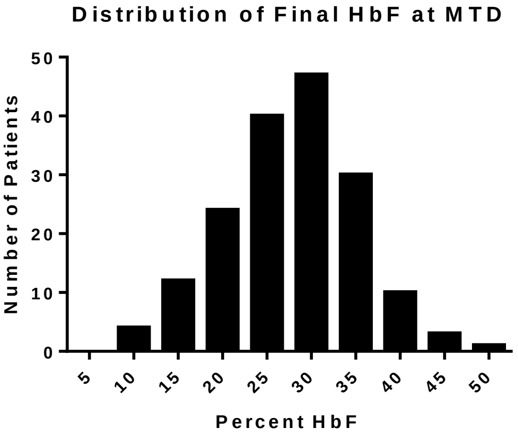

B.

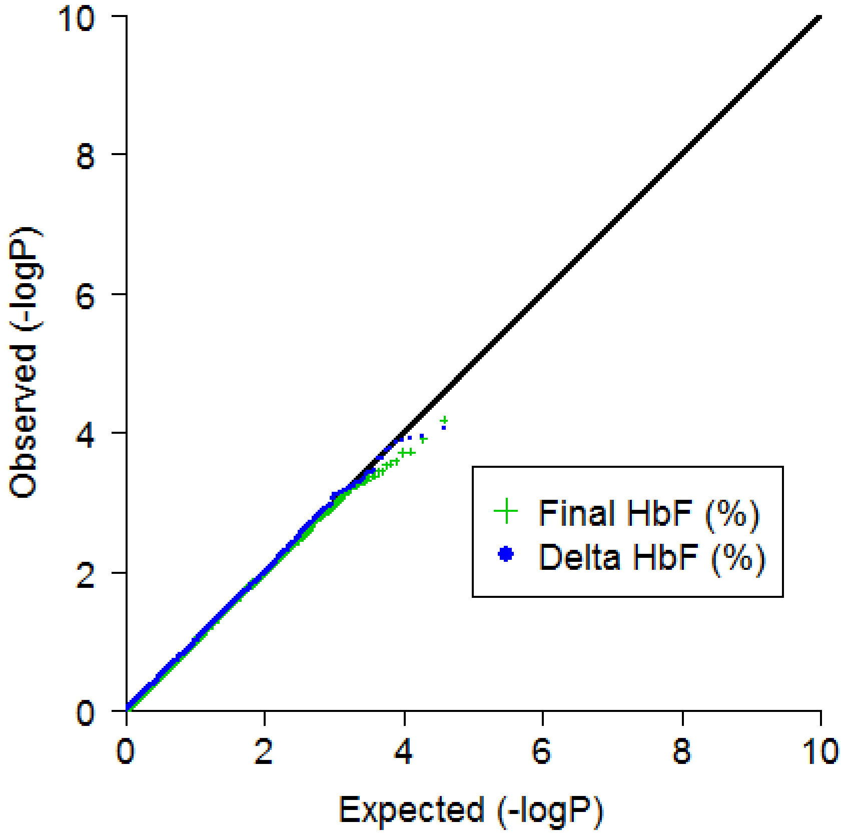

Supplemental Figure 2: Q-Q plot of p-values from WES study of 171 sickle cell patients treated with hydroxyurea. Association between variants and ΔHbF and final HbF at MTD are shown, providing evidence of departure from null distribution.
